# Mitochondrial DNA (mtDNA) haplogroups and serum levels of anti-oxidant enzymes in patients with osteoarthritis

**DOI:** 10.1186/1471-2474-12-264

**Published:** 2011-11-22

**Authors:** Mercedes Fernandez-Moreno, Angel Soto-Hermida, Sonia Pertega, Natividad Oreiro, Carlos Fernandez-Lopez, Ignacio Rego-Perez, Francisco J Blanco

**Affiliations:** 1 Osteoarticular and Aging Research Lab. Rheumatology Division. INIBIC-Hospital Universitario A Coruña. Spain; 2 Epidemiology Unit, INIBIC-Hospital Universitario A Coruña. Spain; 3 CIBER-BBN. ISCIII, Spain

## Abstract

**Background:**

Oxidative stress play a main role in the initiation and progression of the OA disease and leads to the degeneration of mitochondria. To prevent this, the chondrocytes possess a well-coordinated enzymatic antioxidant system. Besides, the mitochondrial DNA (mtDNA) haplogroups are associated with the OA disease. Thus, the main goal of this work is to assess the incidence of the mtDNA haplogroups on serum levels of two of the main antioxidant enzymes, Manganese Superoxide Dismutase (Mn-SOD or SOD2) and catalase, and to test the suitability of these two proteins for potential OA-related biomarkers.

**Methods:**

We analyzed the serum levels of SOD2 and catalase in 73 OA patients and 77 healthy controls carrying the haplogroups J, U and H, by ELISA assay. Knee and hip radiographs were classified according to Kellgren and Lawrence (K/L) scoring from Grade 0 to Grade IV. Appropriate statistical analyses were performed to test the effects of clinical variables, including gender, body mass index (BMI), age, smoking status, diagnosis, haplogroups and radiologic K/L grade on serum levels of these enzymes.

**Results:**

Serum levels of SOD2 appeared statistically increased in OA patients when compared with healthy controls (p < 0.001). Even in those OA patients with higher OA severity (K/L grade IV), the serum levels of this antioxidant enzyme appeared more significantly increased than in OA patients with lower K/L grade (p < 0.001). The mtDNA haplogroups showed an influence on serum levels of catalase (p = 0.054), being carriers of the mtDNA haplogroup J those who showed higher serum levels than non-J carriers (p = 0.057).

**Conclusions:**

The increased levels of SOD2 in OA patients indicate an increased oxidative stress OA-related, therefore this antioxidant enzyme could be a suitable candidate biomarker for diagnosis of OA. Mitochondrial haplogroups significantly correlates with serum levels of catalase

## Background

Osteoarthritis (OA), the most common joint disease and cause of musculoskeletal disability in elderly people, is characterized by late-onset degeneration of articular cartilage, leading to joint destruction and severe impairment of mobility [1]. It is also the main cause of work incapacity and one of the most common reasons for visiting primary physicians. The metabolic and structural changes that take place in the articular cartilage, including the oxidative stress, are thought to play a main role in the initiation and progression of this disease.

Actually, OA-related molecular biomarkers are being developed with the aim of detecting the progression of OA with more reliability and sensitivity, preferably early in the disease process [2,3]. Because of their greater sensitivity compared with radiographs, several molecular markers for bone, cartilage and synovial have been described as useful for the early identification of OA and of patients at high risk for progression, for monitoring disease progression, and for assessing therapeutic response [3-6]. In this sense, our group has recently added new candidate genetic biomarkers, the mitochondrial DNA (mtDNA) haplogroups, which we suggest can be useful as complementary factors when the classical OA-related molecular biomarkers are analyzed.

The mtDNA haplogroups have been associated not only with several multifactorial diseases [7-9] and ageing [10,11], but also with a lower prevalence and severity of knee and hip OA [12,13]. Besides, they modulate the serum levels of some collagen type-II molecular biomarkers [14] as well as some proteolytic enzymes, such as metalloproteinases [15]. The proposed mechanism relies on the different metabolic characteristics of these haplogroups, reflected by the performance of the mitochondrial oxidative phosphorylation system (OXPHOS) of each haplogroup [7,16]. The OXPHOS system is a mitochondrial metabolic pathway that uses energy released by the oxidation of nutrients to produce adenosine triphosphate (ATP) as electrons are transferred from electron donors to electron acceptors, such as oxygen, in redox reactions. These redox reactions, carried out by a series of protein complexes within mitochondria, release energy which is used to form ATP.

The different metabolic characteristics make haplogroup J carriers to show not only lower serum levels of catabolic OA-related biomarkers when compared with those carriers of the haplogroup H [14] but also lower oxygen consumption and lower oxidative damage [17].

As mentioned above, elevated levels of oxidative stress occur in OA and aged cartilage [18]. Although reactive oxygen species (ROS) are involved in the control of various aspects of biological processes in chondrocytes as intracellular second messenger molecules [19], elevated production of ROS leads to i) telomere instability and downregulation of chondrocyte function [20], ii) increased inflammatory response [21], iii) cartilage degradation, by cleaving collagen and aggrecan and activating matrix metalloproteinases (MMPs) [22], and iv) cell death [23]. Oxidative stress also results in the degeneration of mitochondria, the main source of ROS, leading to a leakage of oxidative chain and significant damage to the mitochondrial genome and reduced mtDNA capacity for repair [24].

To prevent an accumulation of ROS-mediated damage, chondrocytes possess a well-coordinated enzymatic antioxidant system formed principally by superoxide dismutases (SODs), catalase and glutathione peroxidase (GPX). SOD2 and SOD3 has been shown to be downregulated in OA cartilage [25-27]; and catalase activity was shown to be increased in OA patients compared with healthy controls [28].

In summary, taking into account the incidence of the oxidative stress in the OA process, as well as the incidence of the mtDNA haplogroups in both the performance of the OXPHOS system and in the OA disease, even on serum levels of classical OA-related molecular biomarkers and proteolytic enzymes, the aim of this work is to perform a retrospective study to evaluate the effects of the mtDNA haplogroups on serum levels of SOD2 and catalase, as well as to test the suitability of these two proteins for potential OA-related biomarkers. The study population is a previously described cohort of OA patients and healthy controls from the north of Spain, consisted in 73 OA subjects and 77 healthy controls carrying the mtDNA haplogroups J, U or H [14].

## Methods

### Subjects

The population analyzed in this study has been described previously [14]. A total of 73 unrelated patients diagnosed with knee or hip OA (25 carrying haplogroup J, 25 carrying haplogroup U and 23 carrying haplogroup H) were included in the present study. Patients meeting the inclusion criteria for this study included individuals of both sexes (52 females; 21 males), older than 41-years-old (mean age: 67.74 ± 8.96 years-old; range: 51-95), and diagnosed with OA following the American College of Rheumatology (ACR) criteria [29]. Knee and hip radiographs from 148 subjects were classified according to Kellgren and Lawrence (K/L) scoring from Grade 0 to Grade IV [30]. Only those joints of OA patients diagnosed with OA were examined for presence of radiographic OA. Of the 77 subjects who met the inclusion criteria for normal subjects, 25 carried haplogroup J, 25 haplogroup U and 27 haplogroup H. These control subjects included 39 females and 38 males older than 41-years-old (mean age: 66.01 ± 11.88 years-old; range: 42-94), who did not meet the ACR criteria for knee or hip OA. In all cases, informed consent and the agreement of the ethical committee from Galician Health Administration were obtained.

### mtDNA haplogroups genotyping

The samples obtained for the study were haplogroup-typed using a previously described assay [12]. Briefly, a multiplex PCR for the amplification of 6 mtDNA fragments that contain 6 informative mtDNA single nucleotide polymorphisms (SNPs) was performed, followed by a single base extension assay (SBE) prior purification of the amplified PCR products. Finally, the fragments were run in an automatic DNA sequencer (ABI 3130XL) to assign the mtDNA haplogroups based on the combination of the informative mtDNA SNPs. For this study, only subjects carrying mtDNA haplogroups J, H or U were included.

### Molecular biomarkers

Fasting blood samples were collected from each subject in plain tubes containing separation gel. These were allowed to stand for 20 minutes, then centrifuged for 10 minutes at 800 g. The serum was then divided into aliquots and stored at -80°C pending assay.

For this study, the serum levels of two antioxidant enzymes were measured: SOD2 and catalase. They were measured in our facilities using enzyme-linked immunosorbent assays (ELISAs) according to the manufacturer's recommendations. Serum SOD2 levels were measured using a kit from ABfrontier (Republic of Korea) and catalase was measured using a Camel catalase ELISA kit from Cusabio Biotech (Wuhan, China).

Determination of the serum levels of these enzymes was performed by simultaneously assaying OA patients and healthy controls, regardless of the mtDNA haplogroup.

### Statistical analysis

Statistical analyses were performed using SPSS software, release 17 (Chicago, USA). In univariate analysis, a non-parametric study was performed utilizing the Mann-Whitney *U*-test to compare serum molecular marker concentrations between OA patients and healthy controls. The Kruskal-Wallis test was used to compare molecular marker concentrations among the three haplogroups (J, U and H) regardless of the diagnosis. To look for possible correlations among the two enzymes analyzed, a correlation analysis using Spearmans Rho was used. In all cases, the Bonferroni correction for multiple comparisons was applied. Thus, p-values were obtained after multiplying the number of outcomes tested by the number of haplogroups (k = 2 × 3).

Following these preliminary analyses, an analysis of covariance (ANCOVA) was used to evaluate the effects on enzyme serum levels (dependent variable) of each of the haplogroups as well as diagnosis, adjusting for the confounder effects of gender, body mass index (BMI), smoking status and age. When a significant effect was found either in haplogroups or in diagnosis, the conservative Bonferroni post hoc multiple comparisons test was performed to compare group means. If the interaction between diagnosis and haplogroup was statistically significant main effects should not be interpreted, so differences among the 2 × 3 = 6 possible combinations of diagnosis (OA and healthy control) and haplogroups (H, J and U) were analyzed by using the same Bonferroni post hoc multiple comparisons test. Before the multivariate analysis, a distribution analysis using the Kolmogorov-Smirnov test showed that the both SOD2 and catalase were not normally distributed; therefore, their concentrations were square and log transformed respectively to obtain a normal distribution. Model diagnostic statistics were also analysed using residual plots.

The influence of radiographic grade to the serum levels of the molecular markers was tested using the non-parametric Jonckheere-Terpstra test for ordered groups. For this approach we obtained the radiographic grade of 148 subjects out of the 150 that are part of the study. Using K/L scores, subjects were divided into three radiographic groups: group A included 77 healthy controls (K/L grade 0 and grade I), group B consisted of 47 OA patients with K/L grades II and III, and group C included 24 OA patients with K/L grade IV.

## Results

### Non-parametric analysis of serum levels of the molecular markers

We first compared the serum levels of the enzymes assayed between OA patients and healthy controls. The results showed that levels of SOD2 were significantly increased in OA patients (p < 0.001) (Table 1). Serum levels of catalase showed a non significant trend toward increased values in OA patients, compared to healthy controls (Table 1).

**Table 1:** Serum levels of molecular markers in healthy controls and osteoarthritis (OA) patients

	Healthy controls (n = 77)		OA (n = 73)		
**Markers**	**Mean (SD)**	**Median**	**Mean (SD)**	**Median**	p*

SOD2 (ng/mL)	20.83 (30.81)	13.50	36.38 (11.16)	36.41	< 0.001**
Catalase (ng/mL)	14.50 (27.68)	5.03	21.88 (40.94)	8.00	0.189

(*) Mann-Whitney non-parametric *U*-test

(**) indicates statistical significance alter Bonferroni correction (p ≤ 0.05)

SD = Standard deviation

Interestingly, the haplogroups have a significant influence on serum levels of catalase regardless of diagnosis, so that carriers of haplogroup J showed higher levels of catalase (p = 0.054). Regarding to SOD2, the results did not reach the statistical significance, but a trend toward decreased serum levels in carriers of haplogroup H compared with non-H carriers was detected (Table 2).

**Table 2:** Serum levels of molecular markers in the mitochondrial DNA (mtDNA) haplogroups H, U and J

	Haplogroup H (n = 50)		Haplogroup U (n = 50)		Haplogroup J (n = 50)		
**Markers**	**Mean (SD)**	**Median**	**Mean (SD)**	**Median**	**Mean (SD)**	**Median**	p*

SOD2 (ng/mL)	23.27 (13.92)	24.88	31.34 (34.52)	26.29	30.70 (19.60)	30.68	0.149
Catalase (ng/mL)	21.20 (37.49)	5.22	8.37 (11.34)	4.37	25.14 (45.42)	8.57	0.054**

(*) Kruskal-Wallis non-parametric test

(**) indicates statistical significance alter Bonferroni correction (p ≤ 0.05)

SD = Standard deviation

When we compared the three groups based on the K/L score, only SOD2 showed a significant trend towards proportionally higher serum levels in both groups B (K/L grade II and III) and C (K/L grade IV) than in group A (K/L grade 0 and I) (p < 0.001) (Table 3). Finally, no significant correlations among the serum levels of these two enzymes were detected.

**Table 3:** Demographics of the study population and serum levels of enzyme markers grouped by radiological severity (Kellgren/Lawrence Score) and distribution of the haplogroups H, U and J in the 3 radiographic groups

	Healthy controls (n = 77)		OA patients (n = 73)					
Mean age (range)	66.01 years-old (42-94)		67.74 years-old (51-95)					
Females/Males	39 females/38 males		52 females/21 males					

**Haplogroups**	**Group A (n = 77)** K/L grade 0 and I		**Group B (n = 47)** K/L grade II and III		**Group C (n = 24)** K/L grade IV			

Haplogroup H, n(%)	25 (32.5)		18 (38.3)		7 (29.2)			
Haplogroup U, n(%)	25 (32.5)		12 (25.5)		13 (54.2)			
Haplogroup J, n(%)	27 (35)		17 (36.2)		4 (16.7)			

**Markers**	**Mean (SD)**	**Median**	**Mean (SD)**	**Median**	**Mean (SD)**	**Median**	**J-T**	p*

SOD2 (ng/mL)	20.83 (30.81)	13.50	35.75 (10.44)	35.02	38.10 (12.74)	39.23	7.796	< 0.001**
Catalase (ng/mL)	14.50 (27.68)	5.03	22.82 (42.49)	8.56	20.58 (38.87)	8.00	1.227	0.220

Group A: K/L grade 0 and I; Group B: K/L grade II and III; Group C: K/L grade IV

(*) Jonckheere-Terstra (J-T) non-parametric test

J-T = established J-T statistic

(**) indicates statistical significance after Bonferroni correction (p ≤ 0.05)

SD = Standard deviation

### Multiple regression analysis

We then performed a multiple regression analysis to assess the effects of the mtDNA haplogroups and clinical variables including gender, age, BMI, smoking status and diagnosis on the serum levels of the enzymes analyzed.

The results obtained for SOD2 showed that serum levels of this antioxidant enzyme were significantly increased in OA patients compared to healthy controls (p < 0.001). On the contrary, no significant influence of the mtDNA haplogroups neither significant interactions between mtDNA haplogroups and diagnosis were detected (Table 4).

**Table 4:** p values from the ANOVA table for the analysis of covariance of serum levels of the two enzymes analyzed

Variable	SOD2	Catalase
Gender	0.422	0.536
Age	0.800	0.211
BMI	0.655	0.471
Smoking status	0.785	0.084
Diagnosis	< 0.001*	0.249
Haplogroups:		
Haplogroup H (vs non-H)	0.133	0.894
Haplogroup J (vs non-J)	0.580	0.057*
Haplogroup U (vs non-U)	0.354	0.075
Diagnosis X Haplogroup	0.488	0.883

(*) indicates statistical significance (p ≤ 0.05)

The results of the multiple regression analysis for catalase showed that serum levels were slightly modulated by the mtDNA haplogroup J, bordering on the statistical significance, so that carriers of this haplogroup have higher levels than non carriers (p = 0.057) (Table 4). On the contrary, carriers of the mtDNA haplogroup U showed a non-significant trend towards lower levels of catalase than non-U carriers (p = 0.075) (Table 4). However, as described for SOD2, no significant interactions between mtDNA haplogroups and diagnosis were found.

## Discussion

In this study we analyzed whether some biomarkers related to the oxidative stress are modulated by the mtDNA haplogroups in the context of the OA disease. The choice of the mtDNA haplogroups J and U was due to their role in the prevalence and severity of knee and hip OA [12,13]; the mtDNA haplogroup H was also selected because is the most frequent mtDNA haplogroup in European populations [31].

Both SOD2 and catalase are two of the main members of the well-coordinated enzymatic antioxidant system in chondrocytes since ROS are, besides matrix metalloproteinases, the main biochemical factors of cartilage degradation [32]. Briefly, SOD2 catalyses the dismutation of superoxide anion (O_2_^-^) to oxygen (O_2_) and hydrogen peroxide (H_2_O_2_), and this H_2_O_2 _in turn is eliminated by catalase, the most efficient enzyme for the degradation of H_2_O_2_, or by glutathione peroxidase. An imbalance in this coordinated system will lead to the increased oxidative stress that takes place in the OA disease.

mtDNA haplogroups have been described to be involved in the development of the OA disease [12,13], as well as in the modulation of the serum levels of classical collagen type-II molecular biomarkers [14] and proteolytic enzymes [15], probably due to their different OXHPOS performance [7,16] and the differences in the oxidative damage and oxygen consumption among them [17]. These particular characteristics make that some of these haplogroups show a lower ROS production and apoptosis [33,34], hence their involvement on serum levels of certain antioxidant proteins is a matter of relevance.

The results obtained in this work showed that SOD2 was significantly increased in OA patients, even those OA patients with higher radiographic grade showed the highest values too; meanwhile catalase showed a trend toward higher levels in the OA group, but did not reach the statistical significance. The results obtained also showed a influence of the mtDNA haplogroups on serum levels of catalase, so that those carriers of the mtDNA haplogroup J showed higher levels of catalase than non-J carriers regardless of diagnosis.

Despite some authors showed a downregulation of SOD2 in OA cartilage [27,35], the increased serum levels of this enzyme in OA patients showed in this work reflect an increased oxidative stress OA-related, as described by other authors [36]; besides, the deleterious effects of increased SOD2 have been supported in various studies [37]. However, serum levels of catalase were non-significantly increased in OA patients, notwithstanding the fact that catalase has greater stability than SOD2 [28,32]; on the contrary, catalase was increased in carriers of haplogroup J, showing a different pattern than for SOD2.

Despite the influence of the haplogorup J on serum levels of catalase, the potential role of the mtDNA haplogroups on serum levels of these two antioxidant enzymes seems slight, contrarily to the observed in both collagen type II biomarkers and proteolytic enzymes [14,15]. Hence, a future approach will be to analyze the serum levels of these two antioxidant enzymes in a prospective larger cohort of samples to ascertain their increased serum levels in OA patients and therefore to validate their suitability as candidate OA biomarkers, and even their modulation by the mtDNA haplogroups.

Attending to the "BIPED" (Burden of disease, Investigative, Prognostic, Efficacy of intervention and Diagnostic) biomarker classification [38], SOD2 could be considered as a diagnostic marker pending validation, and also a burden of disease marker since, as shown in this study, its serum levels are significantly different attending to the K/L score. Regarding to the mtDNA haplogroups, they could be considered as investigative markers because, although their combination with some of the OA-related biomarkers seems to influence their behaviour, to increase information and replicate the findings to allow inclusion into one of the existing categories is still necessary. Otherwise, because we did not find significant differences in serum levels of catalase between OA patients and healthy controls, we consider this potential biomarker as another investigative one.

Biochemical markers of cartilage degradation have received much attention in comparison with others; overall, uCTX-II and sCOMP had the best performance of all currently biochemical markers, they were investigated most frequently and broadly, and scored in the higher ranges of scores form most "BIPED" categories; however, none of the current biochemical markers is sufficiently discriminating to aid diagnosis and/or prognosis of OA, attending to a systematic review carried out by Spil van and collaborators [39].

This study show some limitations that must be considered. This is a retrospective study in which the selection of the samples was based on the availability of serum samples carrying the mtDNA haplogroups J, U or H with no criterion when obtaining the serum samples; in this sense, it must be pointed out that, for serological analysis, the standardization of sample collection may improve biochemical marker performance since diurnal rhythms and influences of exercise have been described for several biochemical markers [40,41]; besides, repeated thawing and freezing must be avoided in order to obtain reliable results. Otherwise, the sample size may be small when multiple analysis are performed, and specially if we compare with larger GWAS studies, although this work is not. Hence, more samples must be analyzed to replicate these findings, and therefore we have initiated a new prospective collection of samples to try to replicate the findings showed in this study.

## Conclusions

To our knowledge, this is the first work that correlates the serum levels of these enzymes with the mtDNA haplogroups in the context of the OA disease. In summary, this work highlights the significantly increased serum levels of SOD2, an important antioxidant enzyme, in OA patients, demonstrating the existence of oxidative stress. Besides, serum levels of catalase are slightly increased in carriers of the mtDNA haplogroup J, when compared with non-J carriers. As described for both type II collagen biomarkers and some proteolytic enzymes OA-related, the influence of the haplogroups on the serum levels of catalase could arise from the different metabolic characteristics of these mitochondrial variants, that make some of them showed a different performance of the OXPHOS system [7,16]. Similarly, since serum levels of SOD2 are significantly decreased in healthy controls and significantly increased in OA patients with K/L grade IV, the use of SOD2 as a candidate biomarker for diagnosis of OA is proposed. However, a new prospective study must be performed in order to replicate these findings.

## Competing interests

The authors declare that they have no competing interests.

## Authors' contributions

MFM carried out the experimental procedures of the mitochondrial haplogroups identification. She helped to draft the manuscript and he has given final approval of the version to be published. ASH carried out the quantification of SOD-2 and catalase by ELISA. SP performed the statistical analysis and helped to draft the manuscript. NO and CFL collected the samples and checked clinical histories for the inclusion and exclusion criteria. IRP has been involved in the conception and design of the study, he helped to draft the manuscript and he has given final approval of the version to be published. He was also involved in the mitochondrial haplogroups identification. FJB conceived the study, and participated in its design and coordination and helped to draft the manuscript. All authors read and approved the final manuscript.

## Pre-publication history

The pre-publication history for this paper can be accessed here:

http://www.biomedcentral.com/1471-2474/12/264/prepub
